# A randomized clinical trial of ascorbic acid in open abdominal aortic aneurysm repair

**DOI:** 10.1186/s40635-015-0050-5

**Published:** 2015-07-01

**Authors:** Martin J Duffy, Cecilia M O’Kane, Michael Stevenson, Ian S Young, Denis W Harkin, Brian A Mullan, Daniel F McAuley

**Affiliations:** Regional Intensive Care Unit, Royal Victoria Hospital, Belfast, UK; Centre for Infection and Immunity, The Queen’s University Belfast, First Floor, Health Sciences Building, 97 Lisburn Road, Belfast, BT9 7BL UK; Centre for Public Health, The Queen’s University Belfast, Belfast, UK; Regional Vascular Surgery Unit, Royal Victoria Hospital, Belfast, UK

**Keywords:** Ascorbic Acid, Ichaemia-Reperfusion injury, Abdominal aortic aneurysm, Perioperative anaesthesia, Perioperative critical care

## Abstract

**Background:**

Open AAA repair is associated with ischaemia-reperfusion injury where systemic inflammation and endothelial dysfunction can lead to multiple organ injury including acute lung injury. Oxidative stress plays a role that may be inhibited by ascorbic acid.

**Methods:**

A double blind, allocation concealed, randomized placebo-controlled trial was performed to test the hypothesis that a single bolus dose (2g) of intra-operative parenteral ascorbic acid would attenuate biomarkers of ischaemia-reperfusion injury in patients undergoing elective open AAA repair.

**Results:**

Thirty one patients completed the study; 18 received placebo and 13 ascorbic acid. Groups were comparable demographically. Open AAA repair caused an increase in urinary Albumin:Creatinine Ratio (ACR) as well as plasma IL-6 and IL-8. There was a decrease in exhaled breath pH and oxygenation. Lipid hydroperoxides were significantly higher in the ascorbic acid group following open AAA repair. There were no other differences between the ascorbic acid or placebo groups up to 4 hours after removal of the aortic clamping.

**Conclusions:**

Open AAA repair caused an increase in markers of systemic endothelial damage and systemic inflammation. Administration of 2g parenteral ascorbic acid did not attenuate this response and with higher levels of lipid hydroperoxides post-operatively a pro-oxidant effect could not be excluded.

**Trial registration:**

ISRCTN27369400

## Background

Open Abdominal Aortic Aneurysm (AAA) repair necessitates the use of an aortic cross-clamp which causes an ischaemia-reperfusion injury to remote tissues which can lead to multiple organ dysfunction syndrome (MODS) including acute lung injury (ALI) and death [1].

Ischaemia-reperfusion injury is associated with an increase in oxidative stress through the accumulation of reactive oxygen species (ROS) which can up-regulate pro-inflammatory pathways contributing to remote organ dysfunction [2,3]. Lipid peroxidation is central in oxidative stress and can be measured using end products [4]. Ischaemia leads to endothelial activation with increased expression of endothelial adhesion molecules [5]. Reperfusion enhances this response and is associated with up-regulation of plasma cytokines [6] and increased leucocyte interaction [7]. Increased urinary albumin:creatinine ratio (ACR) has been associated with impaired endothelial-dependent vasodilation [8], a surrogate measure of endothelial dysfunction [9]. There is evidence of systemic endothelial dysfunction as measured by urinary albumin:creatinine ratio (ACR) in patients undergoing open AAA repair [10] correlating with outcome from ALI in this setting [11,12]. In patients undergoing open AAA repair a rise in ACR measured 4 hours following induction of anaesthesia predicted those at risk of post-operative pulmonary dysfunction [13].

Pulse Wave Analysis (PWA) provides a bedside measure of systemic endothelial function and has been shown to independently predict mortality in the critically ill [14]. Pulmonary dead-space fraction is increased in the acute respiratory distress syndrome (ARDS) and may reflect the extent of pulmonary endothelial dysfunction [15].

Ascorbic acid can act as a reactive oxygen species (ROS) scavenger [16,17]. Low ascorbic acid levels have been described in humans subjected to ischaemia-reperfusion injury during cardiac surgery [18,19] and are associated with acute inflammation [20]. Ascorbic acid attenuates endothelial dysfunction in animal models of acute lung injury (ALI) [21] a condition characterized by uncontrolled inflammation [22]. Ascorbic acid can improve endothelial function [23] by inhibiting apoptosis [24], and preserving endothelial barrier function [25]. As a scavenger it reduces oxidative uncoupling of endothelial nitric oxide synthase (eNOS) leading to further ROS production [26,27]. The hypothesis in this study was that an intra-operative bolus dose (2g) of parenteral ascorbic acid may attenuate markers of oxidative stress, inflammation and endothelial dysfunction caused by open AAA repair.

## Methods

This was a single centre, double blind allocation concealed, randomized placebo-controlled trial. The protocol was approved by the local institution, the UK Medicines and Healthcare products Regulatory Agency and the regional ethics committee. Written informed consent was obtained from each participant prior to study commencement. The trial is registered with www.isrctn.com (ISRCTN27369400).

The study was performed at the Royal Victoria Hospital, Belfast, a tertiary care teaching hospital. Adults undergoing elective open AAA repair were eligible for enrollment. Exclusion criteria were age less than 18 years old, a history of hyperoxaluria or glucose-6-phosphate dehydrogenase deficiency, prior antioxidant therapy, known allergy to ascorbic acid or to anaesthetic agents specified in anaesthetic protocol, participation in another intervention trial within 30 days or a lack of consent.

Patients were randomly assigned to receive intraoperative intravenous ascorbic acid 2 g in 250 ml 0.9% saline or 250 ml 0.9% saline placebo over 15 minutes following induction of anaesthesia. Block randomization was performed by an independent statistician. The randomization assignments were concealed in sealed, tamper-proof envelopes that were opened sequentially by an independent pharmacist. When an eligible subject was recruited, the pharmacist allocated the subject to the designated treatment group, maintaining blinding. Ascorbic acid and 0.9% saline were prepared by the independent pharmacist and had an identical appearance. All staff and participants were blinded to treatment allocation.

### Data collection

Baseline demographic data were collected including medical history. All participants underwent transperitoneal open AAA repair via midline laparotomy. Anaesthesia was provided via a standardized technique.

**Figure 1:**
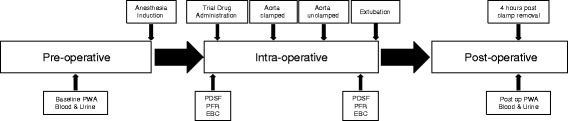
Summary of study period events and measurements. PWA – Pulse Wave Analysis, PDSF – Pulmonary Dead-Space Fraction, PFR – PaO_2_:FiO_2_ Ratio, EBC – Exhaled Breath Condensate.

A summary of events and measurements performed is shown in Figure 1.

### Measurements

Plasma and serum samples were obtained from arterial blood samples taken from the indwelling arterial catheter. Blood samples were centrifuged at 2000 rpm for 10 minutes at 10°C and then samples were frozen at -80°C until analysis. Urine samples were collected and frozen at -80°C until analysis.

Plasma Von Willebrand Factor (vWF) level was measured by ELISA as previously described [11]. Soluble forms of Intercellular adhesion molecule (sICAM)-1, vascular cell adhesion molecule (sVCAM)-1, endothelial (sE)-Selectin and platelet (sP)-Selectin were measured using a Fluorokine® MAP multiplex kit (R&D Systems, Minneapolis, MN, USA) with a BioRad BioPlex™ analyser (Luminex Corp., Austin, TX, USA). Interleukins IL-6 and IL-8 were measured using a cytometric bead array (R&D Systems, Abingdon, UK) as previously described [28]. Serum highly sensitive C Reactive Protein (hsCRP) analysis was performed using the Quantex CRP ultra sensitive quantitative system (ILab Chemistry Systems, Instrumentation Laboratory Company, Lexington, Mass). Serum aqueous phase lipid hydroperoxides were measured using the ferrous iron/xylenol orange (FOX) assay as previously described [29]. Plasma 8-iso-Prostaglandin F2α was measured using a direct enzyme immunoassay kit (Assay Designs Inc., Ann Arobor, Michigan, USA) with a BioRad BioPlex™ analyser (Luminex Corp., Austin, TX, USA). To measure plasma ascorbic acid, plasma was mixed with 5% meta-phosphoric acid (MPA) to stabilise ascorbic acid and then measured on a Cobas FARA centrifugal analyser with a fluorescent attachment according to Vuilleumier & Keck [30]. To determine urinary ACR, albumin was measured in urine using a commercially available immunoturbidimetric assay containing antibody specific for human albumin (Randox Laboratories Ltd. Crumlin, Co. Antrim, UK). Creatinine was measured using a modified Jaffe method as previously described (Roche Diagnostics, IN, USA) [31].

PWA was used to assess systemic endothelial function as previously described [14]. This was performed with the SphygmoCor Mx System (AtCor Medical, Sydney, Australia). An indwelling 20G radial arterial catheter was used to obtain a calibrated peripheral arterial pressure waveform. The central aortic waveform was derived from the peripheral arterial waveform using a validated transfer function [32,33]. Aortic augmentation is defined as the difference between the first systolic peak (caused by left ventricular contraction) and second systolic peak (caused by wave reflection) and the augmentation index (AIx) is this difference expressed as a percentage of the central pulse pressure. AIx is calculated and corrected to a heart rate to 75 beats/min. The AIx following nebulised salbutamol 2.5mg measures endothelium dependent vasodilation (EDV) [34].

Pulmonary Dead-Space Fraction (Vd/Vt) was measured using volumetric capnography utilizing the NICO2 Monitor (Philips Respironics UK Ltd., Tangmere, UK) to calculate the partial pressure of expired CO_2_. This is then used in the Enghoff modification of the Bohr equation [35]. This method has been validated in a cohort of patients with acute lung injury [36].

Exhaled Breath Condensate (EBC) pH as a measure of alveolar neutrophilic inflammation was measured in EBC collected using an RTube™ (Respiratory Research, Austin, TX, USA) as previously described [37]. Exhaled breath condensate pH was measured using an Orion 9803BN micro pH electrode and digital pH meter (Thermo Scientific, Waltham, MA, USA) immediately after collection.

### Statistics

Proportions were used as descriptive statistics for categorical variables. Mean (standard deviation) or median (interquartile range) were used as appropriate after testing for normality with the Kolmogorov-Smirnov test. Between group categorical variables were compared using Fishers exact test and continuous variables with an unpaired Student’s *t*-test or Mann-Whitney *U* test as appropriate. Comparison of variables before and after open AAA repair was made using a paired Student’s *t*-test or Wilcoxon matched pairs test as appropriate. Statistical analysis was performed and graphs generated using Prism 5 for Mac OS X, version 5.0b (GraphPad Software Inc.). A P value of <0.05 was considered significant.

### Power calculation

The sample size calculation was determined using data from a previous study examining plasma vWF concentration in elective open AAA repair patients [12]. Based on these data, mean (standard deviation) vWF concentrations of 175(56) and 125(40) U/dl were assumed for patients receiving placebo and ascorbic acid respectively. On this basis 31 patients were required to complete the study. On the basis of assumed unequal variance between the groups, 18 patients were allocated to receive placebo and 13 to receive ascorbic acid. This provided 80% power to detect a statistical difference if the true difference was as suggested above. This calculation assumed that a two-tailed *t*-test at a 5% significance level was applied.

## Results

**Figure 2:**
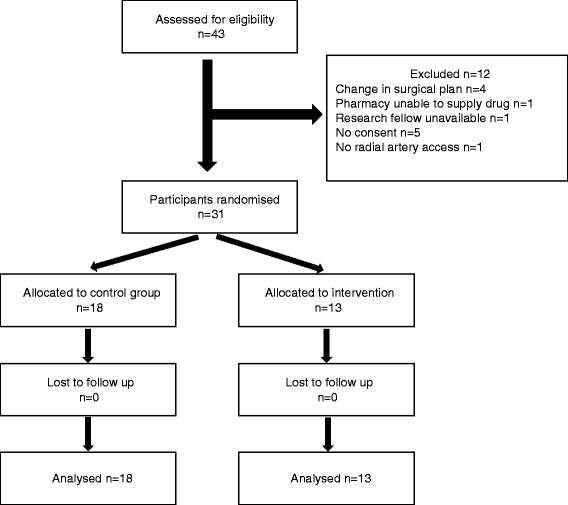
Consort diagram of study recruitment.

Forty three patients were screened over an 18 month period. Twelve patients were excluded. Thirty-one participants received study drug, completed the study and were included in the analysis. Thirteen patients received ascorbic acid and 18 received placebo (Figure 2).

Baseline characteristics were similar in both groups. The duration of ischaemic injury caused by the aortic clamp was similar between groups (Table 1). Mean arterial pressure fell intra-operatively from 101 (17) mmHg to 85 (15) mmHg in the ascorbic acid group (p = 0.03) and from 101 (16) mmHg to 82 (15) mmHg in the placebo group (p < 0.001) but there were no differences between groups either pre- or post-operatively. Ascorbic acid levels measured four hours following removal of the aortic clamp were higher in the ascorbic acid group compared to placebo (104.4 (84.8, 132.7) μmoll-1 vs 18.2 (6.2, 34.5) μmoll-1, p < 0.001).

**Table 1:** **Demographic, historical and pre-operative physiological parameters**

	**Placebo group**	**Ascorbate group**
	**n = 18**	**n = 13**
	**Mean (SD), Median (IQR) or n (%)**	**Mean (SD), Median (IQR) or n (%)**
**Demographics**		
Age (years)	70.4 (7.4)	73.2 (5.9)
Height (m)	1.71 (0.09)	1.71 (0.05)
Weight (kg)	77.1 (11.1)	78.2 (7.7)
BMI (kgm^−2^)	26.4 (3.3)	26.8 (2.8)
Gender (% male)	17 (94%)	11 (85%)
**Past medical history**		
IHD	8 (44%)	4 (31%)
Atrial Fibrillation	0 (0%)	2 (15%)
Hypertension	16 (89%)	12 (92%)
Cerebrovascular Disease	1 (6%)	1 (8%)
**Cardiovascular risk factors**		
Smoking	4 (22%)	5 (38%)
Hypercholesterolaemia	10 (56%)	6 (46%)
Hypertension	16 (89%)	12 (92%)
Diabetes	1 (6%)	0 (0%)
**Drug history**		
Statin	12 (67%)	9 (69%)
ACE Inhibitors	5 (28%)	3 (23%)
β Blockers	7 (39%)	6 (46%)
Diuretics	2 (11%)	3 (23%)
Anticoagulants	5 (28%)	5 (38%)
**Baseline physiological parameters**		
Respiratory Rate (min^−1^)	14 (12,15)	12 (12,15)
Temperature (°C)	36.6 (0.33)	36.8 (0.38)
Heart Rate (min^−1^)	63 (10.3)	60 (13.7)
Peripheral SBP (mmHg)	145.2 (24.9)	150.5 (29.6)
Peripheral DBP (mmHg)	77 (12.4)	76 (11.7)
**Haematological and biochemical indices**		
Haemoglobin (gdl^−1^)	13.7 (1.2)	13.7 (1.1)
Leucocyte Count (x10^9^ml^−1^)	7.3 (1.7)	7.3 (1.8)
Platelet Count (x10^9^ml^−1^)	201 (54)	211 (64)
Urea (mmoll^−1^)	7.8 (2.1)	6.7 (1.8)
Creatinine (μmoll^−1^)	96 (31)	101 (30)
**Surgical details**		
Ischaemic Time (mins)	60.5 (16.9)	64.6 (17.6)

SD, Standard Deviation, IQR, Interquartile Range, BMI, Body Mass Index, SBP, Systolic Blood Pressure, DBP, Diastolic Blood Pressure, IHD, Ischaemic Heart Disease, ACE, Angiotensin Converting Enzyme.

### Effects of ascorbic acid on endothelial function

**Figure 3:**
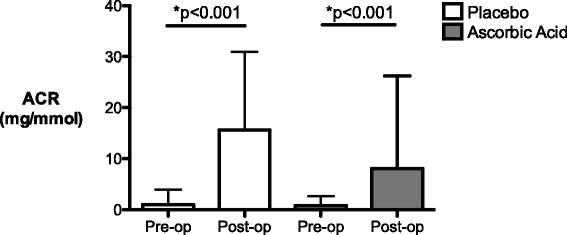
There was a significant increase in urinary ACR in the placebo group from 0.99(0.58,3.9) to 15.6(6.0,30.9)mg/mmol and in the treatment group from 0.77(0.5,2.6) to 8.1(3.1,26.3)mg/mmol. There were no differences between groups either pre- (p = 0.56) or postoperatively (p = 0.37).

Plasma VWF levels were similar between groups both before and after open AAA repair. Similarly there were no differences in EDV between groups either pre- or post-operatively (Table 2). Urinary ACR was significantly higher in both the placebo (p < 0.001) and ascorbic acid (p = 0.01) groups after AAA repair, however there were no differences between the groups (Figure 3). There were no significant changes in pulmonary deadspace following open AAA repair (Table 2).

**Table 2:** **Comparison of biomarkers in ascorbic acid and placebo groups pre- and post-operatively**

	**Pre operative parameters**			**Post operative parameters**		
	**Placebo group**	**Ascorbate group**	**p-value**	**Placebo group**	**Ascorbate group**	**p-value**
	**n = 18**	**n = 13**		**n = 18**	**n = 13**	
	**Mean (SD),**	**Mean (SD),**		**Mean (SD),**	**Mean (SD),**	
	**Median (IQR)**	**Median (IQR)**		**Median (IQR)**	**Median (IQR)**	
**Endothelial function**						
vWF (% Control)	141 (61)	139 (60)	0.92	158 (78)	169 (65)	0.68
EDV (%)	3.5 (1.8, 5.3)	3.0 (1.5, 5.5)	0.42	6.0 (2.5, 8.5)	4.0 (2.0, 6.0)	0.32
Vd/Vt	0.56 (0.06)	0.54 (0.05)	0.53	0.56 (0.06)	0.54 (0.06)	0.74
**Systemic and pulmonary inflammatory mediators**						
Serum hsCRP (mgL^−1^)	2.14 (0.97, 5.75)	1.83 (1.15, 4.49)	0.95	2.85 (1.45, 4.63)	1.96 (1.13, 4.23)	0.27
Arterial pH	7.38 (0.04)	7.40 (0.05)	0.43	7.32 (0.08)	7.29 (0.06)	0.20
**Pulmonary function**						
PaO_2_:FiO_2_ ratio (kPa)	59.6 (47.8, 81.1)	59.6 (46.5, 97.7)	0.44	45.3 (28.3, 49.4)	42.5 (32.7, 51.4)	0.79

SD, Standard Deviation, IQR, Interquartile Range, EDV, Endothelium-dependent vasodilatation, vWF, von Willebrand Factor, Vd/Vt, Pulmonary Deadspace Fraction, EBC, Exhaled Breath Condensate, CRP, C Reactive Protein.

### Effects of ascorbic acid on soluble adhesion molecules

**Figure 4:**
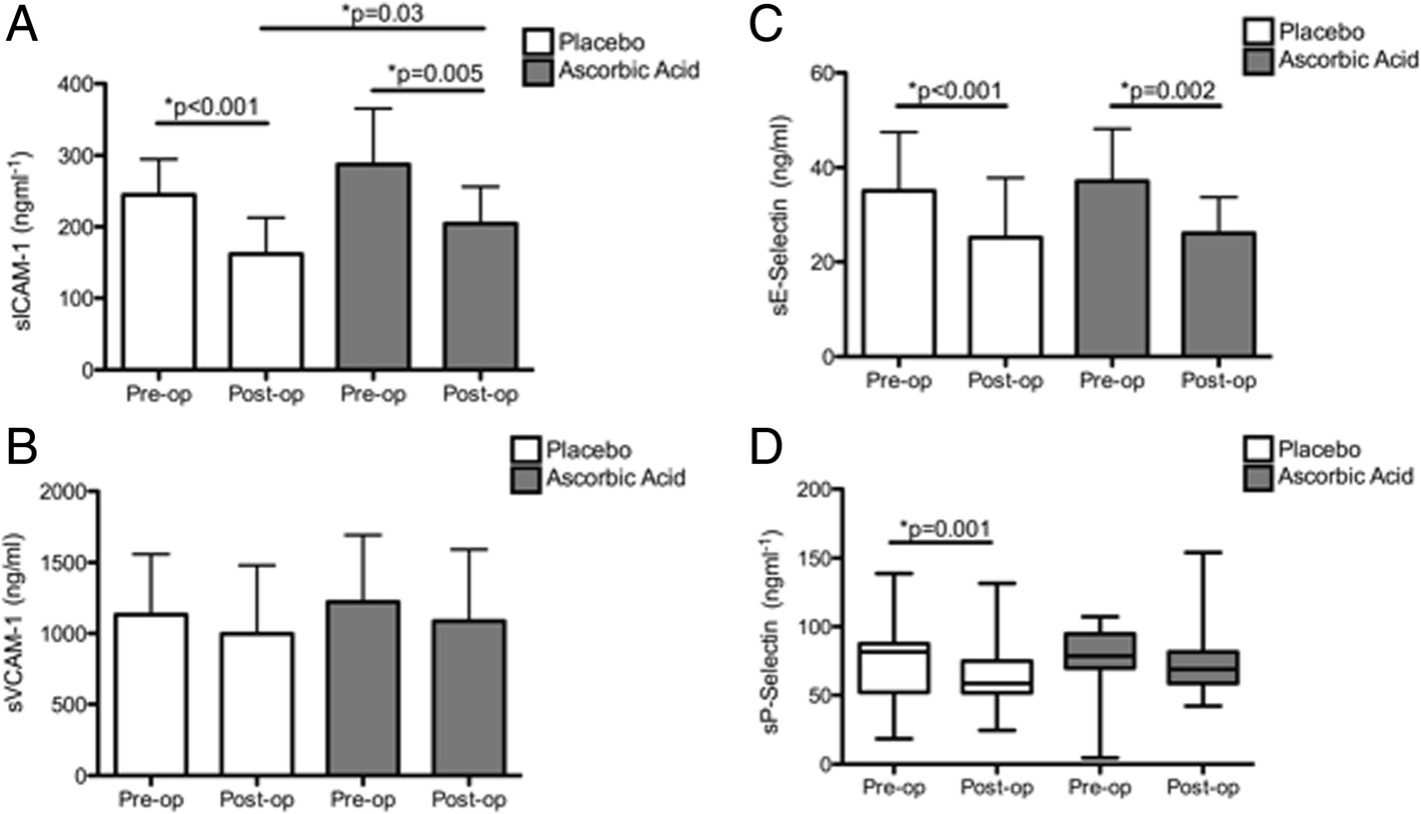
Endothelial adhesion molecules. (**A**) Plasma sICAM-1 levels decreased in the placebo group from 245(50) to 162(51) ng/ml and in the ascorbic acid group from 287(79) to 204(52) ng/ml after AAA repair and post-operatively were significantly lower in the placebo group compared to ascorbic acid (p = 0.03). (**B**) Plasma sVCAM-1 levels did not change after AAA repair. In the placebo group the preoperative level was 1132(427) ng/ml and post-operatively 998(484)ng/ml (p = 0.3). In the ascorbic acid group the preoperative level was 1223(471)ng/ml and postoperatively 1088(505)ng/ml (p = 0.4). There were no differences between groups and any time point. (**C**) sE-Selectin levels decreased in the placebo group from 35(12) to 25(13)ng/ml (p < 0.001) and in the ascorbic acid group from 37(11) to 26(8)ng/ml (p = 0.002) after AAA repair. There were no differences between groups. (**D**) sP-Selectin levels decreased in the placebo group from 82(52,88) to 59(52,75)ng/ml (p = 0.001) after AAA repair. In the ascorbic acid group the levels were similar pre- and post-operatively (79(70,95) vs. 69(59,82)ng/ml) (p = 0.4).

Plasma sICAM-1 fell within both the placebo (p < 0.001) and ascorbic acid (p = 0.005) groups after open AAA repair, There was no difference between placebo and ascorbic acid groups pre-operatively (p = 0.1) however plasma sICAM-1 was higher in the ascorbic acid group post-operatively (p = 0.03 vs placebo, Figure 4A). Plasma sVCAM-1 was similar pre and post open AAA repair in both groups and there was no difference between groups (Figure 4B). Plasma sE-Selectin fell post-operatively within both the placebo (p < 0.001) and ascorbic acid (p = 0.002) groups, although there was no difference between the groups (Figure 4C). Plasma sP-Selectin fell post-operatively within the placebo (p = 0.0001) but not the ascorbic acid (p = 0.41) groups, although there was no difference between the groups (Figure 4D).

### Effects of ascorbic acid on systemic and pulmonary markers of inflammation

**Figure 5:**
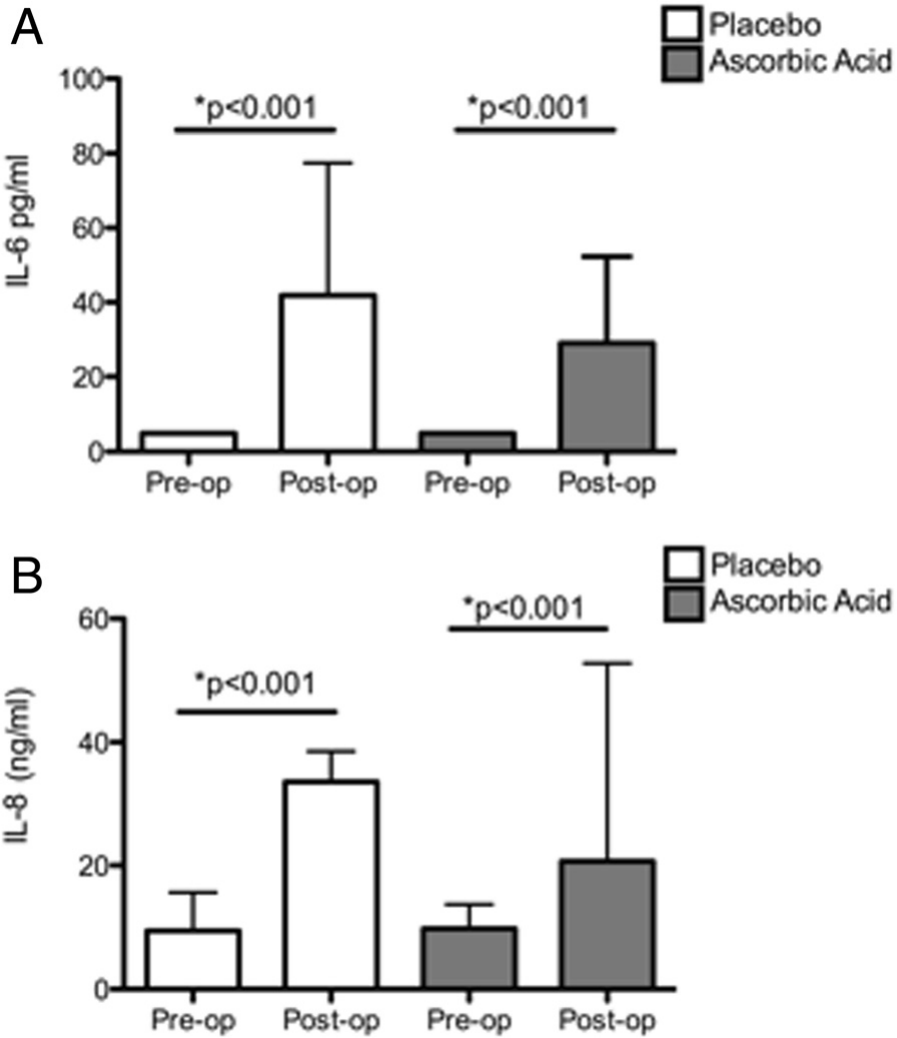
(**A**) IL-6 increased in the placebo group from 4.9(4.9,5.0) to 41.8(33.5,77.4) pg/ml (p < 0.001) and in the ascorbic acid group from 4.9(4.9,5.0) to 29.0(13.3,52.3) pg/ml (p < 0.001) after AAA repair. (**B**) IL-8 levels increased in the placebo group from 9.5(5.6,15.6) to 33.6(18.3,38.6) pg/ml (p < 0.001) and in the ascorbic acid group from 9.8(3.7,13.7) to 20.7(14.8,52.9) pg/ml (p < 0.001) after open AAA repair. There was no difference between groups at any time-point for either of these inflammatory mediators.

Plasma CRP was similar pre and post open AAA repair in both groups and there was no difference between groups (Table 2). Plasma IL-6 increased during the study period in the placebo (p < 0.001) and ascorbic acid (p < 0.001) groups although there was no difference between the groups (Figure 5A). Similarly plasma IL-8 also increased significantly in both placebo (p < 0.001) and ascorbic acid (p < 0.001) groups but again there was no difference between the groups (Figure 5B).

**Figure 6:**
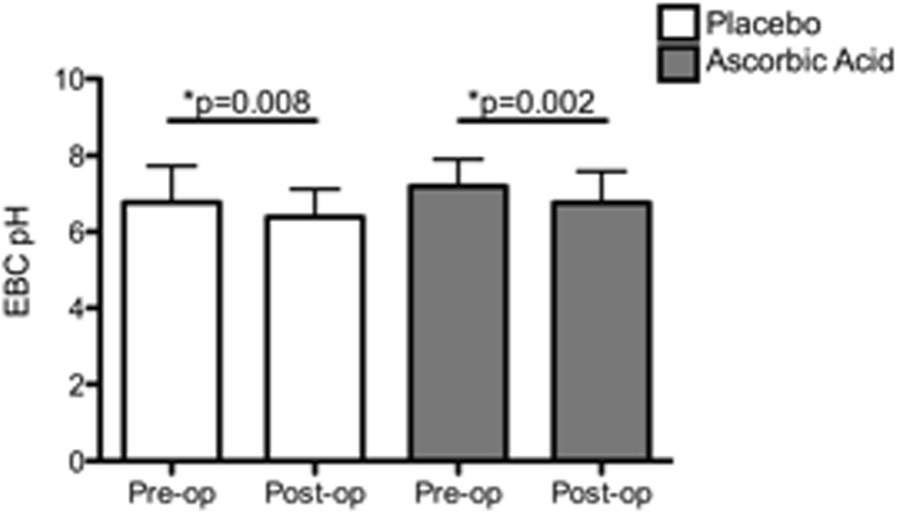
EBC pH decreased in the placebo group from 7.38(0.04) to 7.32(0.08) (p = 0.008) after open AAA repair. In the ascorbic acid group the EBC pH fell from 7.40(0.05) to 7.29(0.06) (p = 0.002) post-operatively. The pre- and post-operative levels were similar between groups (Table 2).

EBC pH decreased in both the placebo (p = 0.008) and ascorbic acid (p = 0.002) groups post-operatively although there was no difference between the groups (Figure 6). Systemic arterial pH also decreased in both the placebo (p < 0.001) and ascorbic acid (p = 0.005) groups post-operatively although there was no difference between the groups (Table 2). There was no significant correlation in the change in systemic arterial pH and EBC pH in either the placebo (p = 0.52, r^2^ = 0.03) or ascorbic acid (p = 0.23, r^2^ = 0.13) groups.

### Effects of ascorbic acid on systemic oxidative stress

**Figure 7:**
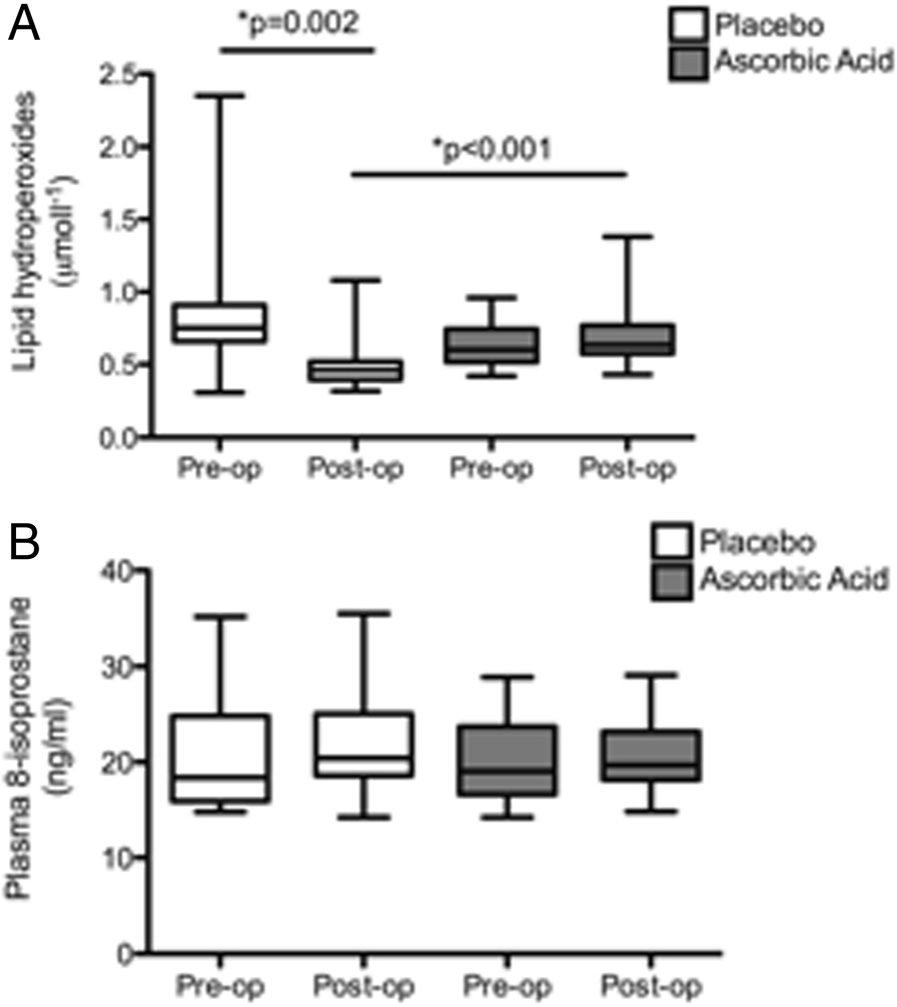
Oxidative stress markers. (**A**) Lipid hydroperoxide decreased in the placebo group from 0.8(0.7,0.9) to 0.5(0.4,0.5)μmol/l (p = 0.002) after AAA repair. In the ascorbic acid group the preoperative level 0.6(0.5,0.7)μmol/l did not change and postoperatively was 0.6(0.6,0.8)μmol/l (p = 0.4). Post-operatively lipid hydroperoxide levels were significantly higher in the ascorbic acid group compared with placebo (p < 0.001). (**B**) Plasma 8-isoprostane levels did not change after AAA repair in either group and there was no difference between groups at any time-point. In the placebo group the preoperative level 18.4(15.9,24.8)ng/ml did not change after AAA repair being 20.4(18.6,25.1)ng/ml (p = 0.3). In the ascorbic acid group the preoperative level 19.1(16.6,23.7)ng/ml did not change and postoperatively was 19.7(18.2,23.2)ng/ml (p = 0.6). There was no difference between groups at any time point.

Plasma lipid hydroperoxides decreased in the placebo group (p = 0.002) but not in the ascorbic acid group (p = 0.38). Furthermore post-operative lipid hydroperoxides were significantly higher in the ascorbic acid group (p < 0.001, Figure 7A). Plasma 8-isoprostane was similar pre and post open AAA repair in both groups and there was no difference between groups (Figure 7B).

### Effects of ascorbic acid on pulmonary function and clinical outcomes

**Figure 8:**
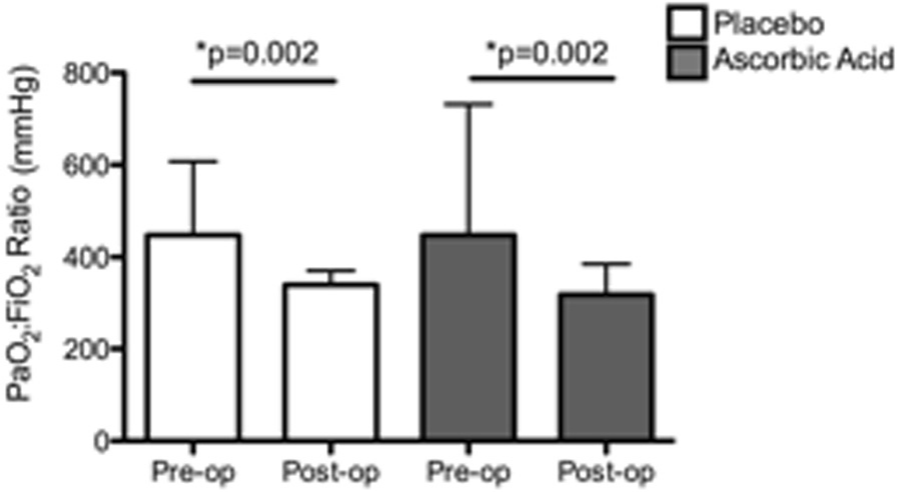
The PaO2:FiO2 ratio decreased in the placebo group from 447 (398, 608) to 340 (212, 370) mmHg (p = 0.002) and in the ascorbic acid group from 447 (349, 733) to 319 (245, 385) mmHg (p = 0.002) after AAA repair. There were no differences demonstrated between groups (Table 2).

The PaO_2_:FiO_2_ ratio fell significantly in both placebo (p = 0.002) and ascorbic acid (p < 0.001) groups although there was no difference between groups (Figure 8).

Post-operative infections were diagnosed in 3 (17%) of the placebo group and in 2 (8%) of the ascorbate group (p = 0.62). Only 3 patients in each group required post-operative invasive ventilation and the duration of ventilation was similar between groups (p = 0.77). The duration of stay in critical care was similar between the placebo and ascorbic acid groups (2.9 (3.5) days versus 2.3 (3.2) days, p = 0.64). Similarly there was no difference in hospital stay (10.2 (4.8) versus 8.8 (2.6) days, p = 0.37). There was one post-operative fatality in the placebo group. No serious adverse reactions related to the study drug occurred.

## Discussion

We have found that open AAA repair caused significant changes in markers of systemic endothelial dysfunction as well as an increase in systemic and pulmonary inflammation, and a reduction in a marker of pulmonary function. However ascorbic acid therapy did not attenuate changes in these biomarkers following open AAA repair.

Our findings are in keeping with previous data showing increased urinary ACR and inflammation and with a decrease in PaO_2_:FiO_2_ ratio following open AAA repair [12,38]. In contrast to these findings however we did not see an increased serum CRP or increased plasma vWF. An increasing proportion of participants now receive treatment for co-morbid conditions such as statins. Statins have anti-inflammatory effects [28,39] and may modulate endothelial function modifying responses to open AAA surgery. We found no increase in pulmonary dead space fraction. It is possible that the injury was not sufficient to induce pulmonary endothelial dysfunction or that the time to post operative assessment was of insufficient duration. As part of the standardized anaesthetic protocol, maintenance was with sevoflurane which has been shown to protect the endothelium from ischaemia-reperfusion injury [40] and might have further attenuated any potential pulmonary endothelial injury.

The fall in EBC pH as a marker of alveolar neutrophilic inflammation was an interesting finding. This was associated with a decrease in the PaO_2_:FiO_2_ ratio. The fall in EBC pH in this study may reflect pulmonary inflammation given that there was no correlation found with this decrease and changes in systemic pH.

Ischaemia-reperfusion injury has been used to provoke acute lung injury (ALI) in animal models [41]. The obligate ischaemia-reperfusion injury of open AAA repair is associated with post operative pulmonary dysfunction [42]. A recent observational study found that post-operative respiratory failure was associated with increased mortality [43]. Reflecting findings in ALI, we have shown open AAA repair is associated with local alveolar inflammation as well as systemic endothelial dysfunction and inflammation. Elevated plasma IL-6 and -8 levels are predictive of outcome in patients with ALI [44]. As a marker of systemic vascular permeability, urinary ACR has been shown to have an inverse relationship with PaO_2_:FiO_2_ ratio in a cohort of trauma patients [45]. These findings support the use of open AAA repair as a human model of ALI induced by systemic inflammation. However, in the present study mediators implicated in ALI such as vWF [11] were not elevated. It is possible that endothelial injury was not caused to a measurable level. In a murine model of ALI the administration of parenteral ascorbic acid (200 mg/ml) 30 minutes after a septic insult attenuated the inflammatory response and was associated with improved survival, pulmonary function and coagulation [21]. In this study Fisher and colleagues suggested that the protective effects of ascorbic acid are due to its pleotropic actions on multiple pathways and it is possible in the current study, a lack of benefit may be related to the limitations in biomarkers used.

We investigated a single 2 g bolus of intravenous ascorbic acid following induction of anaesthesia in this study. This dose was informed by previous research where pre-treatment with 2 g ascorbic acid was found to modulate the adverse haemodynamic effects of experimentally induced hyperglycaemia [46]. In addition ascorbic acid (2 g) has been shown to prevent hyperglycaemia-induced endothelial dysfunction in healthy human volunteers [47].

In the present study a decrease in plasma adhesion molecules was found. Haemodilution as part of intra-operative management could be potential mechanism for the reduction in soluble adhesion molecules as well as the lack of increase in serum hsCRP. However, as plasma IL-6 and IL-8 both increased, haemodilution is less likely. Co-morbid treatment may also have influenced adhesion molecules expression. Both statins and β blockers may modify adhesion molecules expression. Human coronary endothelial cells treated with nebivolol in vitro have a decrease in VCAM-1, ICAM-1, E- and P-Selectin expression [48]. Interestingly in another study of open AAA repair there was a non-significant fall in both ICAM-1 and VCAM-1 [49]. This has been mirrored in an in vitro study of human aortic endothelial cells which showed exposure of these cells to hypoxia and subsequent reoxygenation did not upregulate surface expression or shedding of adhesion molecules [50].

There was no increase in markers of oxidative stress in the placebo group unlike in other studies. Prosaglandin F_2α_ is increased both on hospital arrival and during the perioperative period in patients with ruptured AAA repair [51]. Malondialdehyde, another marker of oxidative damage was increased at one and 24 hours post open AAA repair [52]. In a recent small observational study derivatives of reactive oxygen metabolites were used to define post-operative oxidative stress in patients undergoing aortic surgery. This showed that in those having open AAA repair this marker was no different at 24 hours after surgery, but was significantly elevated 1 week later [53]. This study had an earlier sampling time, older cohort of patients and aortic clamp times contributing to the different data between studies.

Ascorbic acid did not improve markers of endothelial function or attenuate the inflammatory response markers in this study. In previous research pretreatment with 2000mg parenteral ascorbic acid prevented arterial stiffness secondary to induced hyperglycaemia in healthy male volunteers [23]. In a study of 37 critically ill patients with burns the use of high dose ascorbic acid (66 mg/kg/hr) for 24 hours reduced fluid requirements, wound oedema, and severity of pulmonary dysfunction [25]. In a double-blind randomized placebo-controlled trial of 216 critically ill patients, enteral ascorbic acid (500 mg/day) and tocopherol (400 IU/day) was associated with a decrease in 8-isoprostane. The anti-oxidant treated group had improved 28-day mortality and more ventilator free days [54]. We found lipid hydroperoxides were increased with ascorbic acid. This increase in lipid peroxides was not seen in plasma 8-isoprostanes, another measure of oxidative stress. Lipid hydroperoxides can be affected by dietary factors not accounted for in the current study. Isoprostane levels however, are not affected by diet [55] and may be a better measure of oxidative stress [56]. Previous studies utilizing the FOX assay have indicated its lack of specificity for hydroperoxides [57]. However, in keeping with our findings, Bailey at al found 2000 mg ascorbic acid orally two hours before operation in patients undergoing major vascular surgery increased both lipid peroxides and plasma IL-6 [58]. Possible reasons for increased oxidative stress markers in this current study may relate to the timing of sampling and range of ischaemic times. It is possible that at the dose used in this study, ascorbic acid acted as a pro-oxidant with no beneficial effects on either the endothelium or measured mediators of inflammation.

There are numerous limitations in this study. The patient cohort was small increasing the risk of a type 2 error. Within the participants neither aneurysm size or anatomical location were described. Duration of aortic clamping was recorded but the position was not noted. Although the surgical approach and anaesthetic technique was standardized the quantities of transfused blood products and fluids were not compared. Similarly the need for cardiovascular support was not quantified. These confounding factors may have had a consequence on the resultant endothelial injury. Having taken measurements using a limited number of biomarkers of organ dysfunction up to only 4 hours following aortic clamping may increase the risk of a type 1 error.

## Conclusion

In conclusion, open AAA repair caused increased markers of systemic endothelial dysfunction and inflammation with an associated fall in oxygenation. These findings support the use of open AAA repair as a human model of ALI induced by systemic inflammation. Ascorbic acid administration did not improve the measured biomarkers and limited functional parameters observed.
